# Determination of the Optimal Cutoff Value of Triglyceride That Corresponds to Fasting Levels in Chinese Subjects With Marked Hypertriglyceridemia

**DOI:** 10.3389/fcvm.2021.736059

**Published:** 2021-09-24

**Authors:** Li-Ling Guo, Li-Yuan Zhu, Jin Xu, Ying-Ying Xie, Qun-Yan Xiang, Zhe-Yi Jiang, Yang-Rong Tan, Ling Liu

**Affiliations:** ^1^Department of Cardiovascular Medicine, The Second Xiangya Hospital, Central South University, Changsha, China; ^2^Research institute of Blood Lipid and Atherosclerosis, Center South University, Changsha, China; ^3^Modern Cardiovascular Disease Clinical Technology Research Center of Hunan Province, Changsha, China; ^4^Cardiovascular Disease Research Center of Hunan Province, Changsha, China; ^5^Clinical Nursing Teaching and Research Section, The Second Xiangya Hospital, Central South University, Changsha, China

**Keywords:** triglyceride, marked hypertriglyceridemia, postprandial, cut-off value, daily meal

## Abstract

The level of triglyceride (TG) ≥ 2. 3 mmol/L is suggestive of marked hypertriglyceridemia (HTG) and requires treatment with a triglyceride-lowering agent in high-risk and very high-risk patients as recommended by the 2019 ESC/EAS guidelines for the management of dyslipidemia. However, the optimal cutoff value required to diagnose non-fasting HTG that corresponds to the fasting goal level of 2.3 mmol/L in Chinese subjects is unknown. This study enrolled 602 cardiology inpatients. Blood lipid levels, including calculated non-high-density lipoprotein cholesterol (non-HDL-C) and remnant cholesterol (RC), were measured at 0, 2, and 4 h after a daily Chinese breakfast. Of these, 482 inpatients had TG levels of <2.3 mmol/L (CON group) and 120 inpatients had TG levels of ≥2.3 mmol/L (HTG group). Receiver operating characteristic (ROC) curve analysis was used to determine the cutoff values for postprandial HTG that corresponded to a target fasting level of 2.3 mmol/L. Marked hypertriglyceridemia (≥2.3 mmol/L) was found in 120 (19.9%) patients in this study population. The levels of non-fasting TG and RC increased significantly in both groups and reached the peak at 4 h after a daily meal, especially in the HTG group (*p* < 0.05). The optimal cutoff value of TG at 4 h, which corresponds to fasting TG of ≥2.3 mmol/L, that can be used to predict HTG, was 2.66 mmol/L. According to the new non-fasting cutoff value, the incidence of non-fasting HTG is close to its fasting level. In summary, this is the first study to determine the non-fasting cutoff value that corresponds to a fasting TG of ≥2.3 mmol/L in Chinese patients. Additionally, 2.66 mmol/l at 4 h after a daily meal could be an appropriate cutoff value that can be used to detect non-fasting marked HTG in Chinese subjects.

## Introduction

The 2016 Chinese guideline on the management of dyslipidemia in adults recommends a fasting triglyceride (TG) level of <1.7 mmol/l as ideal, a TG of 1.7~2.3 mmol/L as a borderline increase, and ≥2.3 mmol/L as increased levels of TG, which can be diagnosed as hypertriglyceridemia (HTG) (1). Some scholars have used a higher cutoff value (TG ≥ 2.3 mmol/L) to represent marked HTG (2, 3). In fact, marked HTG (TG ≥ 2.3 mmol/l) is considered to be a more serious condition and is predictive of increased cardiovascular disease (CVD) events (4) even with low-density lipoprotein cholesterol (LDL-C) at goal (5). Therefore, there is a need to reduce non-high-density lipoprotein cholesterol (non-HDL-C) levels to reduce the residual cardiovascular risk in these patients.

Non-HDL-C includes not only LDL-C, but also other atherogenic cholesterol, such as triglyceride-rich lipoproteins (TRL) and their hydrolyzed products [e.g., remnant cholesterol (RC)] (6, 7). Plasma TG mainly exists on TRL, and elevated TG levels represent the increase in TRL and its RC in blood. Remnant cholesterol concentration is highly correlated with the level of triglycerides (8). Therefore, reducing non-HDL-C levels is mainly achieved by reducing TG levels. TG and RC are good predictors of atherosclerotic cardiovascular disease (ASCVD) and are independent risk factors of coronary heart disease (CHD) and myocardial infarction (MI) (9–11). Moreover, TG-lowering agents such as fenofibrate or bezafibrate has been found to be safe and clinically beneficial in statin-treated patients (2, 12, 13).

There is no need of fasting in determining blood lipids (14), and non-fasting blood lipids levels can be routinely assessed at any time of the day after a daily meal (15, 16). However, plasma TG levels are very easily affected by dietary factors and reached a peak at 4 h not only in overweigh patients after a daily meal (17) but also in patients with CHD and hypertension after a high-fat meal (18, 19). This suggests that non-fasting serum TG concentration at 4 h could be used to measure postprandial hypertriglyceridemia. Many large epidemiologic studies have identified non-fasting TG as a more robust marker for arteriosclerotic cardiovascular disease (ASCVD) compared with fasting TG (20, 21). Furthermore, the American Association of Clinical Endocrinologists (AACE), the National Lipid Association (NLA), and the 2019 ESC/EAS guidelines for the management of dyslipidemias all recommended treatment for marked high triglyceride levels (TG > 2.3 mmol/L) with a triglyceride-lowering agent in high-risk and very-high-risk patients (22–24). However, there is no report on the determination of the optimal cutoff value after a daily meal in the diagnosis of HTG that corresponds to the target fasting level of 2.3 mmol/L in Chinese subjects. Therefore, in this study, we aimed to explore the metabolic characteristics of blood lipids in patients with high TG of ≥2.3 and those in control group after a daily breakfast and to determine the non-fasting cutoff value that corresponds to the target fasting TG level of 2.3 mmol/L in Chinese subjects.

## Materials and Methods

### Study Subjects

From March 2017 to July 2020, 602 inpatients were recruited from the Department of Cardiovascular Medicine of the Second Xiangya Hospital, Central South University. Overall, 482 inpatients had TG levels of <2.3 mmol/L (CON group), and 120 inpatients had TG ≥ 2.3 mmol/L before admission (HTG group). Before enrollment, medical history and the use of lipid-lowering drugs were collected. Patients with fasting serum TG concentration of <5.6 mmol/L, LDL-C of <4.1 mmol/L, and TC of <6.2 mmol/L were included in this study. Patients with endocrine disease, digestive disease, autoimmune disease, hepatic and renal diseases, cancer or other serious diseases, or NYHA heart function class III–IV were excluded. This study was approved by the Ethics Committee of the Second Xiangya Hospital of Central South University. All patients signed an informed consent.

### Specimen Collection

All enrolled patients were Chinese Han nationality, and they were fasted for 10 h and ate breakfast in the morning in accordance with the own dietary habits of the subjects. Their meals had high-carbohydrate content, such as rice, noodles, bread, etc. The participants were not allowed to eat any food and exercise too much within 4 h after breakfast, and they can drink a little water.

### Laboratory Assays

The measurement method of blood lipid has been described in detail in previously published literature (17, 25). In short, venous blood sample was drawn in fasting and non-fasting after breakfast and were sent for analysis in a central laboratory for TG, TC, LDL-C, and HDL-C levels. Non-HDL-C and RC levels were estimated using the following formula: non-HDL-C = TC – (HDL-C); RC = TC – (HDL-C) – (LDL-C) (26).

### Estimated Sample Size

The calculation formula of sample size about estimated value was as follows: n1 = n2 = 2 × [(t_α_ + t_β_)s/δ]^2^ (n1 and n2 are the required contents of the two samples respectively; t_α_ and t_β_ are the t values corresponding to inspection level α and type II error probability β, respectively, and s is the estimated value of the overall standard deviation; δ is the difference between the two means). Using two-sample *t*-test, the test efficiency is 90% at the test level of 5% on both sides. The required estimated value of sample size is calculated based on the pre-experimental data of 2 and 4 h after a meal, and the larger value is taken as the sample size required for this study. According to the difference between TG groups at 2 and 4 h after a meal, the calculated sample size is 14 and 35, respectively. Taking the maximum value of each group, about 35 people are needed in each group, and a total of 70 people need to be included in this study. This study included 602 people, 120 in the HTG group and 482 in control group; so the sample size is appropriate.

### Statistical Analysis

Data analysis was performed using the SPSS version 22.0 and Prism 6.0. One-way analysis of variance (ANOVA) for repeated measures within a group or ANOVA for completely randomized measures between groups was used to assess any differences between the means of the variables, as appropriate. Values of numerical characteristics were presented as mean ± standard deviation and were analyzed using the *t*-test, if normally distributed in the two groups. Categorical variables were expressed as percentages and were analyzed by the chi-square test in the two groups. The area under the curve (AUC) and the increment of AUC (iAUC) for measurements of TG and RC concentrations at baseline and after breakfast were estimated using the trapezoidal method (27). Pearson's correlation coefficient calculations were performed to analyze the correlation coefficient (R). Furthermore, the optimal cutoff value for non-fasting TG at 4 h was determined by evaluating the area under the receiver operating characteristic (ROC) curve. All *p*-values were two tailed, and *p* < 0.05 was considered statistically significant.

## Results

### Clinical Characteristics

There were 602 inpatients in our study population, and marked hypertriglyceridemia (≥2.3 mmol/L) was observed in about 19.9% of the participants. The baseline characteristics in the CON group (*n* = 482) and HTG group (*n* = 120) in terms of age, sex, diabetes mellitus, percentage of smoking, and taking statins were well balanced (Table 1). Body mass index in the HTG group was higher than that in the CON group (*p* < 0.05). The proportion of overweight and obese individuals, CHD patients, and the use of acipimox capsules in the HTG group were higher than those in the CON group (*p* < 0.05) (Table 1). Additionally, BMI was positively correlated with serum TG levels both in fasting and non-fasting state (*p* < 0.0001) (Supplementary Figure 1).

**Table 1:** Baseline characteristics of the study population.

	**CON** **(*n* = 482)**	**HTG** **(*n* = 120)**
Age (year, SD)	55.3 ± 12.1	54.9 ± 10.8
Men, *n* (%)	282 (58.5)	80 (66.6)
BMI, kg/m^2^	24.2 ± 3.5	26.2 ± 3.2^*^
OW and OB, *n* (%)	242 (50.2)	94 (78.3)^*^
Current smoking, *n* (%)	188 (39.0)	53 (44.2)
DM, *n* (%)	89 (18.5)	28 (23.3)
CHD, *n* (%)	253 (52.5)	77 (64.2)
Taking statins, *n* (%)	198 (41.1)	57 (47.5)^*^
Taking statins ≥3, *n* (%)	97 (20.1)	22 (18.3)
Ezetimibe, *n* (%)	62 (12.9)	21 (17.5)
Acipimox capsules, *n* (%)	0	4 (3.3)^*^
TC, mmol/L	3.93 ± 0.79	4.20 ± 0.79^*^
LDL-C, mmol/L	2.40 ± 0.69	2.68 ± 0.67^*^
Non-HDL-C, mmol/L	2.84 ± 0.72	3.31 ± 0.71^*^
HDL-C, mmol/L	1.10 ± 0.25	0.89 ± 0.16^*^
TG, mmol/L	1.32 ± 0.46	3.05 ± 0.63^*^
RC, mmol/L	0.44 ± 0.14	0.62 ± 0.17^*^

*HTG group: fasting TG ≥2.3 mmol/L; CON group: fasting TG <2.3 mmol/L. BMI, body mass index; OW and OB, overweigh (BMI 24–27.9) and obesity (BMI ≥ 28); CHD, coronary heart disease; TC, total cholesterol; HDL-C, high-density lipoprotein cholesterol; LDL-C, low-density lipoprotein cholesterol; non-HDL-C, non-high-density lipoprotein cholesterol; TG, triglyceride; RC, remnant lipoprotein cholesterol*.

* *p < 0.05 when compared with the patients in the CON group*.

### The Changes in Non-Fasting Blood Lipids

**Figure 1:**
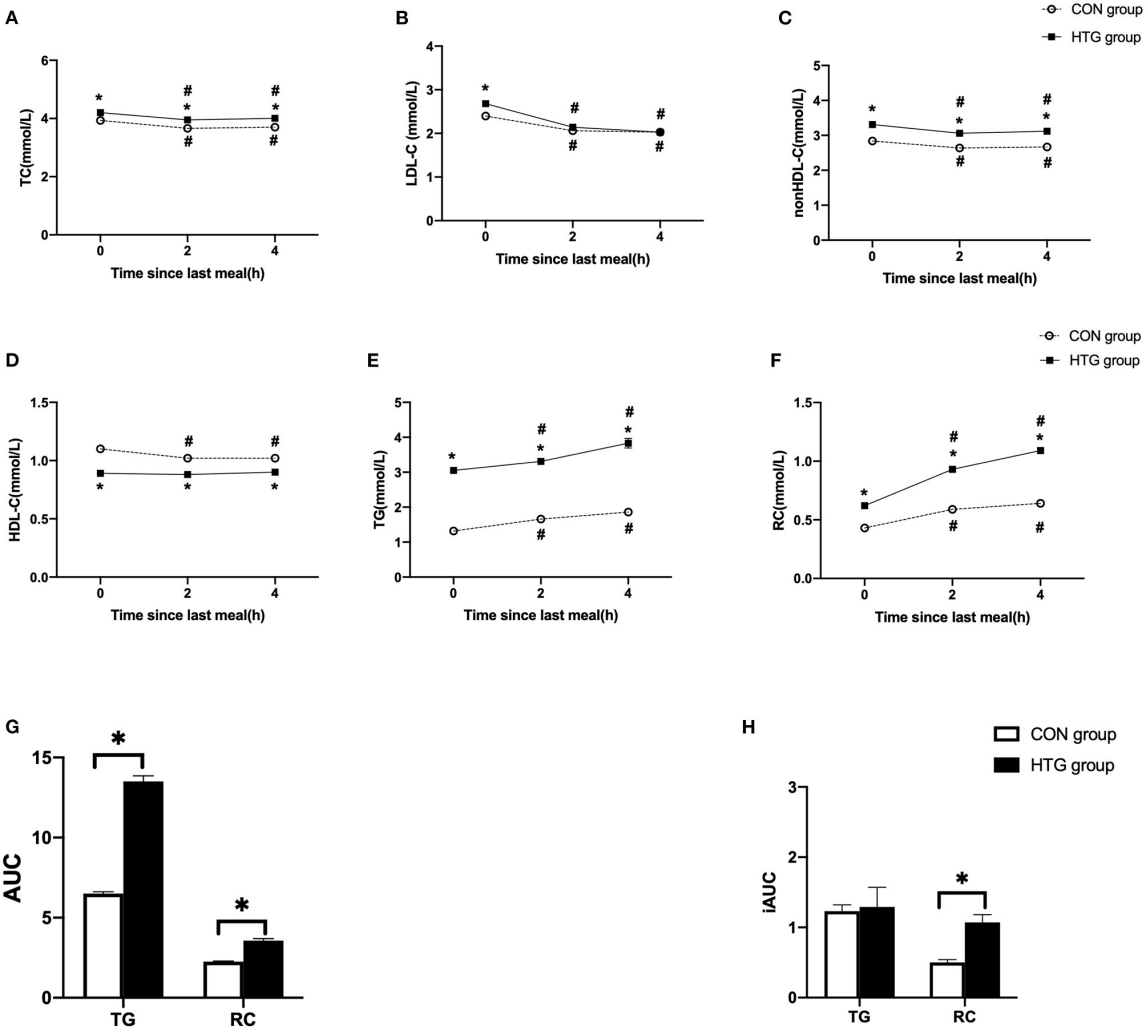
Postprandial changes in blood lipids after a daily meal in two groups. **(A,B,D,E)** The changes in serum concentrations of total cholesterol (TC), low-density lipoprotein cholesterol (LDL-C), high-density lipoprotein cholesterol (HDL-C), and triglyceride (TG). **(C,F)** The changes in serum concentrations of non-HDL-C and remnant cholesterol (RC) determined by calculated methods. **(G)** Comparison of total area under the curve (AUC) for TG and RC between two groups. **(H)** Comparison of increase in AUC (iAUC) for TG and RC between two groups. Hypertriglyceridemia (HTG) group: fasting TG ≥ 2.3 mmol/L. CON group: fasting TG < 2.3 mmol/L. Values are mean ± standard error (SE). One-way analysis of variance (ANOVA) for repeated measures within a group or ANOVA for completely randomized measures between groups was used to assess any differences between the means of the variables, as appropriate. **#***p* < 0.05 when compared with the fasting level in the same group. *p < 0.05 when compared with the patients in the CON group.

The fasting TC, LDL-C, non-HDL-C, TG, and RC levels were significantly higher, and HDL-C levels were lower in the HTG group vs. CON group (Figure 1; Table 1).

After a daily breakfast, the serum concentrations of TC, LDL-C, and non-HDL-C significantly reduced, while those of TG and RC significantly increased (*p* < 0.05). The non-fasting HDL-C levels were significantly decreased at 2 or 4 h vs. fasting value in the CON group (*p* < 0.05), while that in HTG group has no significant differences (Figure 1D). Moreover, the levels of TG and RC peaked in 4 h after a daily breakfast. In the postprandial state, the TC, non-HDL-C, TG, and RC levels at each time point in the HTG group were higher than those in the CON group; however, the concentration of HDL-C was lower in the HTG group than that in the CON group (*p* < 0.05) (Figure 1).

The AUC of TG or RC in the HTG group was significantly higher than that in the CON group (*p* < 0.05). However, only the iAUC of RC in the HTG group was significantly higher than that in the CON group, except for the TG levels (*p* < 0.05, Figures 1G,H).

### The Correlation Between Areas Under the Curve for Triglyceride and Remnant Cholesterol With Blood Lipid Concentration

**Figure 2:**
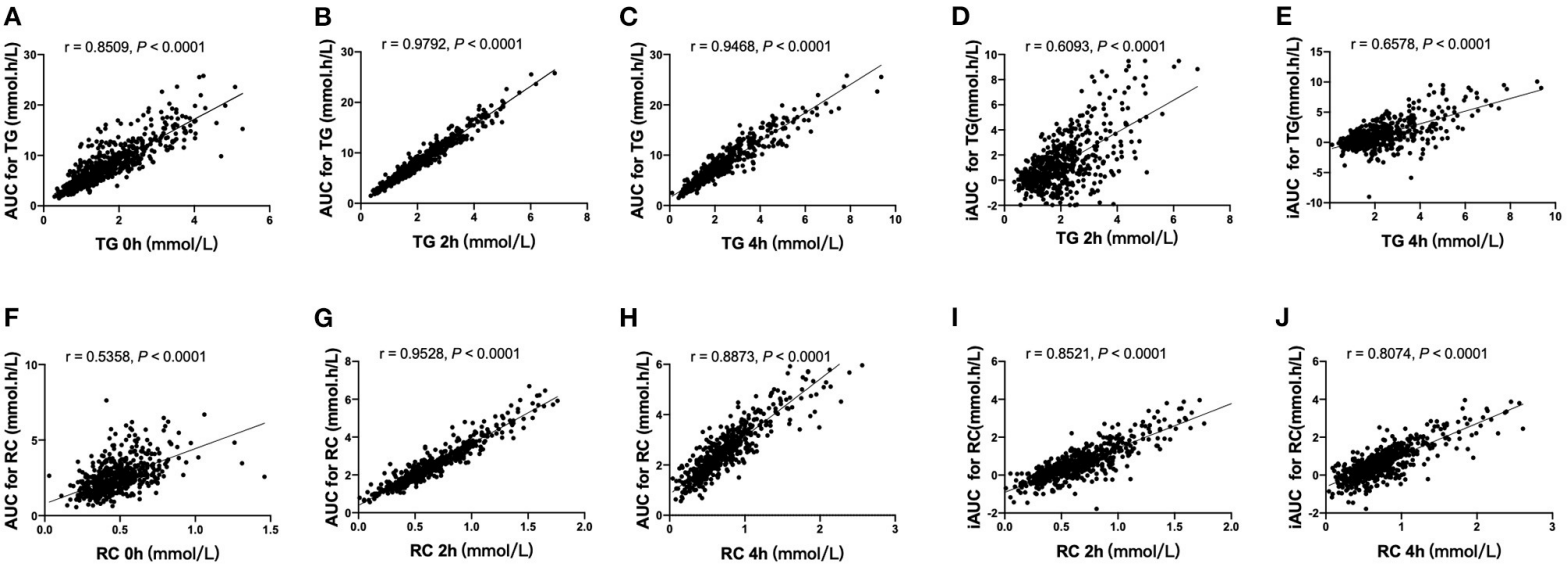
Correlation analysis of AUCs for TG and RC with blood lipid concentration in the fasting and non-fasting state. **(A–C)** The correlation between AUC for TG and serum concentrations of TG in the fasting state **(A)**, at 2 h **(B)** and 4 h **(C)**. **(D,E)** The correlation between iAUC for TG and serum concentrations of TG at 2 h **(D)** and 4 h **(E)**. **(F–H)** The correlation between AUC for RC and serum concentrations of RC in the fasting state **(F)** and at 2 h **(G)** and 4 h **(H)**. **(I,J)** The correlation between iAUC for RC and serum concentrations of RC at 2 h **(I)** and 4 h **(J)**.

Correlation analysis of the total population (*n* = 602) revealed that AUC for TG or RC correlated significantly with fasting and postprandial TG or RC, respectively (*p* < 0.0001). However, iAUC for TG or RC only correlated with postprandial TG or RC (*p* < 0.0001), respectively. The highest correlation coefficient (r) was found between AUC for TG or RC with postprandial TG or RC (TG 2 h, *r* = 0.9792; *p* < 0.0001; TG 4 h, *r* = 0.9468; *p* < 0.0001; RC 2 h, *r* = 0.9528; *p* < 0.0001; RC 4 h, *r* = 0.8873; *p* < 0.0001) (Figure 2).

### Evaluation the Percentage of Hypertriglyceridemia

**Figure 3:**
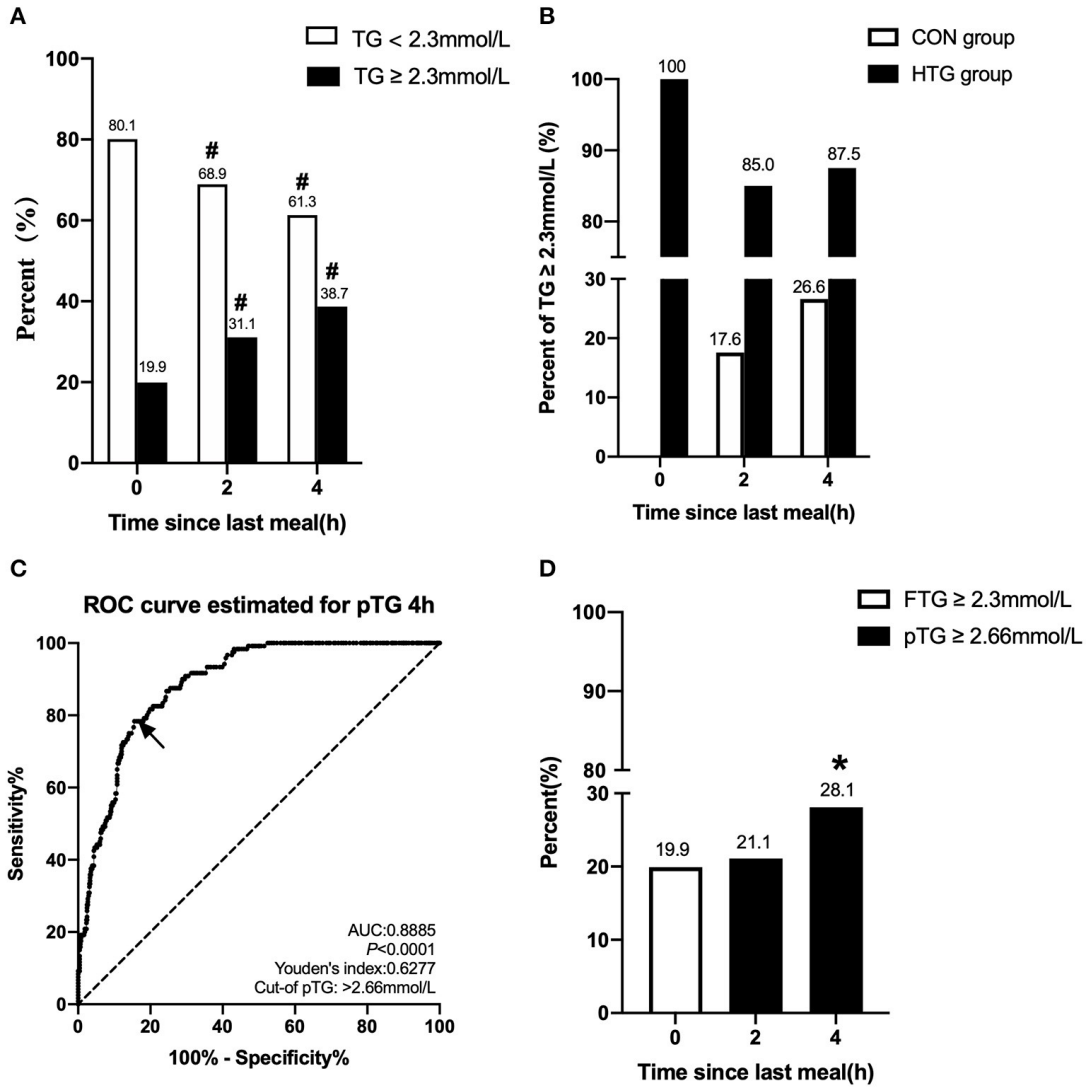
Comparison of the percentage of HTG at different time-points and between different groups. **(A)** Changes in percent of TG ≥ 2.3 mmol/L and <2.3 mmol/L in the fasting state, at 2 and 4 h after a daily meal (*n* = 602). **(B)** Difference in percent of TG ≥ 2.3 mmol/L between patients with fasting TG ≥ 2.3 mmol/L (n = 120) and <2.3 mmol/L (*n* = 482) at three different time-points. **(C)** Receiver operating characteristic (ROC) analysis and Youden's index determined a cutoff point for postprandial TG level at 4 h (pTG 4 h) after a daily meal; the cutoff point was indicated by a solid arrow. **(D)** Comparisons of fasting and postprandial percentages of HTG at different time points according to the new cutoff point. **#***p* < 0.05 when compared with the fasting level in the same group. *p < 0.05 when compared with the fasting level.

We initially evaluated the postprandial TG level based on the fasting cutoff value (TG ≥ 2.3mmol/L) in this study. The incidence of non-fasting HTG at 2 or 4 h was significantly higher than that of fasting value (*p* < 0.05) (Figure 3A). The fasting TG level in the CON group was within normal limits, but when postprandial TG increased, some patients were diagnosed as HTG. However, the proportion of patients with fasting levels of TG ≥ 2.3 mmol/L in the HTG group was 100%, but the level of non-fasting TG in some patients decreased. The percentages of postprandial TG ≥ 2.3 mmol/L at 2 and 4 h in the CON group were 17.6 and 26.6%, respectively, and were 85.0 and 87.5%, respectively, in the HTG group (Figure 3B).

According to the ROC curve analysis, the optimal pointcut value for TG at 4 h to predict HTG was 2.66 mmol/L (sensitivity 78.33%, specificity 84.44%, and AUC 0.8885), and this corresponds to fasting TG ≥ 2.3 mmol/L (Figure 3C). According to the new non-fasting cutoff value, the incidence of postprandial HTG at 2 or 4 h obviously decreased in the study population, 21.1 and 28.1%, respectively, and this was close to the fasting level (19.9%) (Figure 3D).

## Discussion

Globally, hypertriglyceridemia is the most frequent form of dyslipidemia among the general population. Marked hypertriglyceridemia (≥2.3 mmol/L) occurs in about 25% of the patients with metabolic syndrome (2), and this is slightly higher than what was in our study (19.9%). In this study, patients with HTG developed significant non-fasting hyperlipidemia that was characterized by elevated TG levels after a daily meal, especially at 4 h. Furthermore, we observed that patients with higher fasting TG levels (≥2.3 mmol/L) had exaggerated postprandial responses and had higher AUC of TG or RC than those with lower TG concentration (<2.3 mmol/L). More importantly, the non-fasting cutoff value for HTG, 2.66 mmol/L, was first determined using ROC analysis in Chinese patients after a daily meal, and this corresponded to the target levels of TG ≥ 2.3 mmol/L. This study provides a reference value for the diagnosis of postprandial marked HTG in Chinese patients.

Recent studies have shown that an elevated fasting TG level after an oral fat tolerance test is the main factor of an excessive postprandial response in patients with metabolic syndrome (28–30). Now it has been proposed that oral-fat tolerance test (OFTT) is useful to identify postprandial hyperlipidemia in subjects with fasting TG between 1 and 2 mmol/L (89 and 180 mg/dl) because approximately half of them have hidden postprandial hyperlipidemia, which may influence treatment. An OFTT does not provide additional information regarding postprandial hyperlipidemia in subjects with low TG (<1 mmol/L, <89 mg/dl) or increased TG (>2 mmol/L, >180 mg/dl) as fasting TG levels (31, 32). In this present study, 305 patients (about 50%) had TGs of 1–2 mmol/L, and we paid more attention to the non-fasting TG after a daily meal because there was no unified high-fat meal scheme in the world. In recent years, due to the promotion of health education, people are not able to accept a high-fat diet. A considerable number of patients with cardiovascular diseases will actively avoid eating this high-fat diet because of the disease state. Therefore, the patients in our study were not required to eat the specific diet but to eat in accordance with their dietary habits. It was fasting TG, but not free fatty acids or glucose, that showed the most significant correlation with AUC of TG according to linear regression analysis; however, the correlation between non-fasting TG and AUC of TG was not explored (29). Our study showed that the AUC of TG or RC in HTG group was significantly higher than that in the CON group. Furthermore, we also confirmed that AUC for TG or RC correlated significantly with fasting and postprandial TG or RC concentrations, respectively, while iAUC for TG or RC only correlated with postprandial TG or RC, respectively (*p* < 0.0001). Therefore, AUC seems to be a better indicator that reflects the continuous stimulation and even damage caused by TRLs and their remnant cholesterol in the arterial wall during fasting and non-fasting state, while iAUC only represents the postprandial increment of TG or RC after a daily meal. The postprandial state plays a significant role in the development of atherosclerosis (6, 33). Elevated non-fasting triglyceride levels and calculated RC were associated with an increased risk of MI. In the Copenhagen City Heart Study, doubling of non-fasting triglycerides and calculated RC were related to a 1.9- and 2.2-fold remnant risk of myocardial infarction, respectively (11).

According to the 2016 European expert consensus, non-fasting TG concentration should not be >2.0 mmol/L (175 mg/dl) (14); however, according to a scientific statement by the American Heart Association (AHA), it should not exceed 2.26 mmol/L (200 mg/dl) after a daily meal, with a corresponding target fasting goal level of 1.7 mmol/L (34). The new AHA guideline (35) shows that over 175 mg/dl (≥1.97 mmol/L) of TG (fasting or non-fasting) is a risk-enhancing factor in adults 40–75 years of age without diabetes mellitus and 10-year ASCVD risk of 7.5–19.9% (intermediate risk), and this risk-enhancing factor favors the initiation of statin therapy. Our previous study showed that the cutoff value for non-fasting TG level was 2.02 mmol/L after a daily meal for overweight Chinese subjects (17), and this is very close to the 2016 European expert consensus. In a Mexican study, the pointcut value of non-fasting high TG was 3.16 mmol/L after a high-fat meal with 960 kcal in Mexican adults (36), which was very close to our reported cutoff value of high TG (3.12 mmol/L) after a high-fat meal with 800 kcal in our previous study in a Chinese population (37). The similar fat contents (50 vs. 52 g) in both high-fat meals may led to the similarities of two cutoff values for postprandial high TG in these two studies (36, 37). However, the postprandial pointcut value of marked hypertriglyceridemia, which corresponds to a fasting goal level of 2.3 mmol/L, in Chinese subjects after a daily meal or after a high fat meal, was not recommended.

Marked hypertriglyceridemia (≥2.3 mmol/L) was considered to be a more serious condition and was associated with increased CVD events (4), which was supported by the following findings, including the ACCORD Follow-On Study (ACCORDION) (12), Bezafibrate Infarction Prevention Trial (BIP) (13), the FIELD study (2), and the Helsinki Heart Study (HHS) (3). During an average follow-up of 9.7 years in the ACCORDION study, fenofibrate combined with statin therapy was effective to reduce CVD in diabetics with TG levels higher than 204 mg/dl (2.3 mmol/L) and HDL-C lower than 34 mg/dl (0.88 mmol/L). However, fenofibrate failed to show similar benefits in patients with type 2 diabetes without hypertriglyceridemia (12). In the BIP study, the researchers found that bezafibrate may have a significant effect on the treatment of dyslipidemia and CHD in the hypertriglyceridemia subgroup (≥200 mg/dl), and the reduction rate of the primary endpoint was 39.5% (*p* = 0.02), while the primary endpoint decreased by only 7.3% in the total study population (*p* = 0.24) (13). Subjects with markedly elevated triglyceride (≥2.3 mmol/l) had the greatest cardiovascular benefits after fenofibrate treatment, with a 27% reduction in CVD risk in the FIELD study (2). In addition, the incidence of CHD in particularly high-risk subgroups (LDL-C/HDL-C ratio >5 and serum TG > 2.3 mmol/L) can be reduced by approximately 75% in the HHS during gemfibrozil treatment (3). Therefore, it is necessary to combine therapy with drugs to reduce TG in patients with TG ≥2.3 mmol/L and reduce the cardiovascular risk caused by the increase in non-HDL-C as much as possible. According to the 2019 ESC/EAS guidelines for the management of dyslipidemia, triglyceride-lowering agents are recommended to reduce CVD risk in high-risk patients who are at LDL-C goal with TG > 2.3 mmol/L (>200 mg/dl) (24).

People with different diseases will have different reactions after eating a high-fat meal. Our previous studies found that patients with CHD and hypertension had a more obvious increase in TG after a standard high-fat meal, while the control group had only a slight transient increase in TG (19). After a high-fat meal or a daily meal, the TG level gradually increased within 8 h and peaked at 4 h (19, 27, 37). The AUC for TG within 8 h after a meal was used to compare the postprandial fat tolerance of each patient, especially after a high-fat meal, whose postprandial peak concentration contributed the most to AUC for TG. Similarly, Gudmundsson et al. (38) directly detected the serum TG level at 4 h after a meal to reflect the metabolism of postprandial TG. In the total study population, TG peaked in 32.1% of the patients at 2 h and 67.9% of the patients at 4 h after a meal. Among the HTG group (TG ≥ 2.3mmol/L), 25% of the patients reached the peak at 2 h, and 75% of the patients reached the peak at 4 h after a meal. It can be seen from the above that the TG of most patients reaches the peak at 4 h after a meal. Since we are in the postprandial state most of the day, the increase in postprandial TG will be more important than fasting TG (14). If TG was only detected at 2 h, it was possible to miss patients with more obvious increase in TG at 4 h after a meal. Therefore, we chose 4 h after a meal for evaluation in this study.

Since there was no recommendation about the cutoff value of non-fasting-marked HTG, postprandial levels of TG in our present study were preliminarily evaluated based on the fasting cutoff value (TG ≥ 2.3 mmol/L). The percentages of postprandial HTG at each time point after a meal was higher than its fasting value in both the HTG group and CON group (*p* < 0.05), which may be due to the increase in TG caused by daily meals and the inappropriate use of fasting cutoff point of TG. This suggests that the target TG levels of ≥2.3 mmol/L was indeed not suitable for the determination of postprandial HTG in Chinese patients. Therefore, in this study, we preliminarily evaluated the cutoff value of postprandial TG that corresponded to its fasting level after a daily meal. ROC curve analysis was used to determine the optimal cutoff value for postprandial high TG not only in the Women's Health Study (39) but also in overweight Chinese people, after a habitual meal (17). In this study, the cutoff value of non-fasting-marked HTG was 2.66 mmol/L after a daily meal in Chinese patients. When the new cutoff value was used as the evaluation index, the percentage of postprandial HTG in this population was quite close to its fasting values. This suggests that a higher non-fasting TG cutoff point should be used in the evaluation of postprandial marked HTG, and this can fully consider the physiological increase in TG after a daily meal. Therefore, we considered a postprandial TG of 2.66 mmol/L to be as a better cutoff value in Chinese subjects. In the future, we hope to have the opportunity to observe the value of this cutoff point for the diagnosis of postprandial high TG.

There are some limitations in the present study. First, the sample size of this study was small and included patients with complicated cardiovascular diseases. Second, we did not know the exact nutritional consumption of the patients (e.g., proportion of fat and carbohydrate in the meal). However, standardization may lead to an underestimation of the real effect. Third, we did not repeat tests on the blood samples of the same patient, and a single concentration measurement may lead to a regression dilution bias considering the variability of triglyceride concentration. Finally, the blood lipids (especially TG) of hospitalized patients may be affected by the stress of basic diseases or/and lipid-lowering drugs, so it is difficult to say that the cutoff level of non-fasting TG found in the present study can be directly used to evaluate the non-fasting TG status of outpatients who do not receive lipid-lowering drugs. Therefore, it is necessary to carry out similar research on outpatients who do not receive lipid-lowering drugs, which is what we plan to do next.

## Conclusions

In summary, this is the first study to determine the non-fasting cutoff value for fasting TG (2.3 mmol/L) in Chinese patients. It is considered that the concentration of TG at 4 h after a meal (2.66 mmol/L) could be as a suitable cutoff value for detecting postprandial-marked HTG in Chinese patients.

## Data Availability Statement

The original contributions presented in the study are included in the article/Supplementary Material, further inquiries can be directed to the corresponding author/s.

## Ethics Statement

The studies involving human participants were reviewed and approved by the Ethics Committee of the Second Xiangya Hospital of Central South University. The patients/participants provided their written informed consent to participate in this study.

## Author Contributions

LL designed this study. L-LG conducted the study and wrote the paper. L-YZ and JX participated in the collection and analysis of the data. Y-YX, Q-YX, and Y-RT participated in the interpretation of the data. Z-YJ was responsible for collecting blood samples. All authors read the study and approved the manuscript for publication.

## Funding

The study was supported by the National Natural Science Foundation of China (Grant Numbers 81270956 and 81470577).

## Conflict of Interest

The authors declare that the research was conducted in the absence of any commercial or financial relationships that could be construed as a potential conflict of interest.

## Publisher's Note

All claims expressed in this article are solely those of the authors and do not necessarily represent those of their affiliated organizations, or those of the publisher, the editors and the reviewers. Any product that may be evaluated in this article, or claim that may be made by its manufacturer, is not guaranteed or endorsed by the publisher.

## Supplementary Material

Supplementary Figure 1 The correlation between concentrations of TG and BMI in the fasting and non-fasting state. **(A)** The correlation between serum concentrations of TG and BMI in the fasting state. **(B,C)** The correlation between serum concentrations of TG and BMI at 2 h **(B)** and 4 h **(C)** after a daily breakfast. Solid circles represent OW group, and open circles represent CON group.
