# Supplementary figures and images for: Determination of the Optimal Cutoff Value of Triglyceride That Corresponds to Fasting Levels in Chinese Subjects With Marked Hypertriglyceridemia
Source: Front Cardiovasc Med. 2021 Sep 24;8:736059. doi: 10.3389/fcvm.2021.736059 (PMC8498036; doi:10.3389/fcvm.2021.736059)

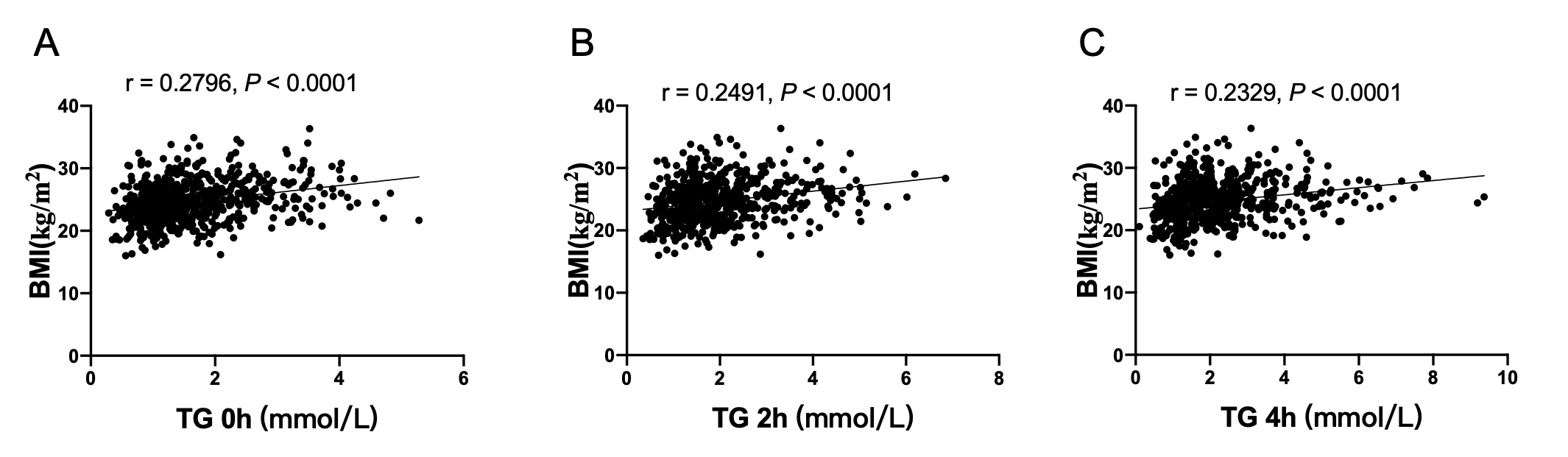

Supplement: Supplementary Figure 1 — The correlation between concentrations of TG and BMI in the fasting and non-fasting state. (A) The correlation between serum concentrations of TG and BMI in the fasting state. (B,C) The correlation between serum concentrations of TG and BMI at 2 h (B) and 4 h (C) after a daily breakfast. Solid circles represent OW group, and open circles represent CON group. [file Image_1.TIFF]
